# Health Tract, No. 162

**Published:** 1863-08

**Authors:** 


HEALTH TRACT, No. 162.

### LEAVING HOME.

When a child leaves home for the first time, after having had the parental eye to watch every footstep, and guard against every danger and harm; when for the first time that ceaseless, sleepless, affectionate care has to be withdrawn, whether that absence is to be for a day, a week, a month, or longer, there is a painful anxiety to give such counsels as may meet the circumstances which are most likely to present themselves. A physician's mind, deeply impressed, as it must be, by the frail tenure of human life; knowing, as he does, the trifling circumstances which frequently put an apparently healthful child in the grave in a few days, labors, not to tell all that may be requisite to insure a safe return, for that would burden the memory, would confuse the mind, or be soon forgotten; but aims to present a few important points, some wide-reaching general principles, or three or four practical facts, which may impress themselves upon the mind and fasten upon the memory of the youngest or most thoughtless.

The thing which may quickest kill, is eating a hearty supper, especially the first one to be taken after reaching the place of destination; for while the journey is sure to give an increased appetite, the bodily exercise and mental excitement connected with it, leave both in a debilitated or exhausted condition; while the thought of being from home, away from father and mother, and more or less among strangers, causes an oppressive depression of spirits, which altogether leaves a person precisely in that condition least capable of resisting very slight causes of disease. Hence, if the stomach is overloaded, and a cold room should be occupied, or an unused bed, or damp sheets, an attack of bilious colic, or uncontrollable diarrhea, convulsions, or fatal pneumonia may very easily destroy life in twenty-four hours. Hence, give a short, clear, succinct injunction:

1st. Never take any thing whatever for supper, while from home at least, but a single cup of weak tea or glass of water, and one piece of cold bread and butter.

2d. Eat only at regular meal-times.

3d. Cut up your food in very small pieces and eat slowly.

4th. Give instant attention to nature's calls, (explaining what these are.)

5th. The moment you cease play or exercise, or come in from walk or ride, go close to the fire for five or ten minutes, if it is fire-time; but if warm weather, spend the same time, or longer, in a closed room, until no perspiration is felt on the forehead.

6th. Never stand a moment in a damp place or retain a damp garment, or sleep near an open door or window.

